# β-hydroxybutyrate and hydroxycarboxylic acid receptor 2 agonists activate the AKT, ERK and AMPK pathways, which are involved in bovine neutrophil chemotaxis

**DOI:** 10.1038/s41598-020-69500-2

**Published:** 2020-07-27

**Authors:** María D. Carretta, Yonathan Barría, Katherine Borquez, Bárbara Urra, Andrés Rivera, Pablo Alarcón, María A. Hidalgo, Rafael A. Burgos

**Affiliations:** Laboratory of Inflammation Pharmacology, Faculty of Veterinary Science, Institute of Pharmacology and Morphophysiology, Universidad Austral de Chile, Valdivia, Chile

**Keywords:** Cell biology, Immunology

## Abstract

Elevated plasma concentrations of the ketone body β-hydroxybutyrate (BHB), an endogenous agonist of the hydroxycarboxylic acid receptor 2 (HCA2), is associated with an increased incidence of inflammatory diseases during lactation in dairy cows. In the early stages of this pathology, an increase in neutrophil recruitment is observed; however, the role of BHB remains elusive. This study characterized the effect of BHB and synthetic agonists of the HCA2 receptor on bovine neutrophil chemotaxis and the signaling pathways involved in this process. We demonstrated that treatment with BHB concentrations between 1.2 and 10 mM and two full selective agonists of the HCA2 receptor, MK-1903 and nicotinic acid, increased bovine neutrophil chemotaxis. We also observed that BHB and HCA2 agonists induced calcium release and phosphorylation of AKT, ERK 1/2 and AMPKα. To evaluate the role of these pathways in bovine neutrophil chemotaxis, we used the pharmacological inhibitors BAPTA-AM, pertussis toxin, U73122, LY294002, U0126 and compound C. Our results suggest that these pathways are required for HCA2 agonist-induced bovine neutrophil chemotaxis in non-physiological condition. Concentrations around 1.4 mM of BHB after calving may exert a chemoattractant effect that is key during the onset of the inflammatory process associated with metabolic disorders in dairy cows.

## Introduction

Neutrophils are the first line of defense against invading microbial pathogens and are an essential arm of the innate immune response in cattle^1^. To enter sites of infection and inflammation, host cells and microorganisms release chemoattractants that direct the migration of neutrophils into the area. Potent chemoattractants for bovine neutrophils include platelet-activating factor (PAF)^2^, complement fraction C5a and interleukin 8 (IL-8)^3^. Chemoattractants bind to specific receptors on the neutrophil plasma membrane that are typically G-protein coupled receptors (GPCRs)^4^. Stimulation of the G protein complex causes the activation of phospholipase Cβ (PLCβ), which results in an increase in intracellular calcium levels^5^. These molecules initiate a cascade of events that produce rapid changes in the cytoskeleton and neutrophil shape, resulting in cell polarization^6^. Several intracellular pathways have been implicated in chemotaxis, such as the mitogen-activated protein kinases (MAPK) ERK1/2, phosphoinositide 3-kinase (PI3K)/Akt and adenosine monophosphate-activated protein kinase (AMPK) α^5,7^ pathways.

Neutrophil chemotaxis contributes to many inflammatory diseases in humans as well in cattle. During the transition to lactation, dairy cows undergo a period of negative energy balance (NEB) that causes an increase in circulatory ketone bodies, predominantly β-hydroxybutyrate (BHB), that can potentially lead to the development of ketosis^8,9^. Subclinical ketosis is defined as an increase in the BHB concentration to ⩾ 1.2 mmol/l in the blood without clinical signs and clinical ketosis is defined when cows have clinical signs regardless BHB levels^10,11^. The onset of subclinical ketosis during the first week of lactation causes great economic impact that is associated with a reduction in milk production and a predisposition to other metabolic and inflammatory diseases^12^. Contradictory data about leukocyte chemotactic ability with ketotic levels of BHB have been shown. Suriyasathaporn et al. (1999) demonstrated that white blood cells from ketotic cows have a lower chemotactic differential than those from nonketotic cows. In contrast, another study showed that leukocytes from ketotic cows obtained by 4-day feed restriction were not impaired in their chemotactic capacity^13^. Therefore, until now, it has been unclear whether bovine neutrophil chemotaxis is altered by BHB and which intracellular signaling pathways are involved in these processes.

It has been proposed that BHB is an endogenous ligand of the G-protein coupled receptor HCA2 (also known as GPR109A or HM74a in humans and PUMA-G in mice)^14^. This receptor was initially identified as the receptor of the antidyslipidemic and antiatherogenic drug nicotinic acid^15–17^ and later as the receptor for the free fatty acid (FFA) butyrate^18^.

Analysis of signal transduction induced by natural HCA2 agonists showed that HCA2 is pertussis toxin-sensitive, indicating that this receptor family couples to G_i_/G_o_-type G proteins^16,17^. Consequently, HCA2 activation results in inhibition of adenylate cyclase activity and a decrease in cyclic adenosine monophosphate (cAMP) levels, as demonstrated in adipocytes^16,17^.

Expression of the HCA2 receptor is found in various immune cells, including human neutrophils, macrophages, dendritic cells and Langerhans cells^19–21^. In neutrophils, HCA2 receptor expression occurs during the late stages of terminal differentiation, since it is not detected in immature bone marrow neutrophils^20^. In mature neutrophils, nicotinic acid through HCA2 has a pro-apoptotic effect, favoring the resolution of inflammation^20^. Besides, nicotinic acid inhibits chemotaxis and proinflammatory cytokine production in mouse macrophages^22^. Also, nicotinic acid potently inhibited human monocyte chemotaxis and adhesion to activated endothelial cells^23^. Therefore, these data suggest that HCA2 could modulate neutrophil chemotaxis.

In addition to nicotinic acid, a series of synthetic compounds based on a pyrazole ring structure were identified as potent full agonists of HCA2 that can activate the receptor on human adipocytes and immune cells^24^. However, there are still no data about the effect of these agonists on bovine cells. In this species, HCA2 is highly expressed in liver and adipose tissue, and analysis of the putative bovine HCA2 sequence suggests that it encodes a functional receptor^25^. Recent data indicate that neutrophil expression of HCA2 is upregulated in animals supplemented with methionine compared to that of unsupplemented control cows^26^, but little is known about HCA2 function in these cells. Therefore, the aim of the present study was to investigate the effect of BHB and other HCA2 agonists on bovine neutrophil chemotaxis and the intracellular signaling involved in this process.

## Results

### BHB induce chemotaxis in isolated bovine neutrophils

**Figure 1:**
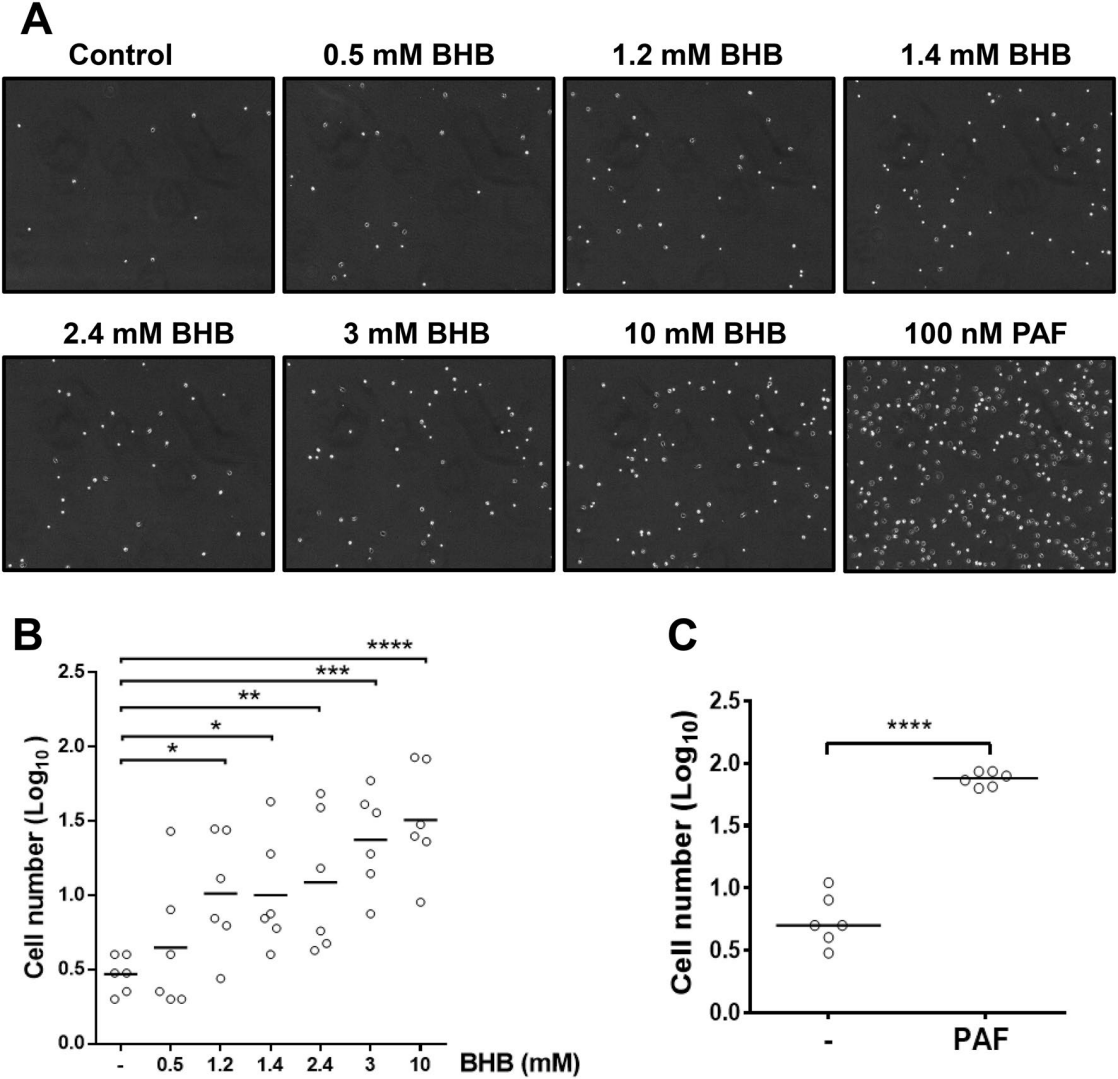
β-hydroxybutyrate induce bovine neutrophil chemotaxis. (**A**) Representative figures of neutrophil chemotaxis. A total of 1 × 10^6^ neutrophils were added to the upper part of the transmigration chamber, and (**B**) β-hydroxybutyrate or (**C**) PAF was added to the lower reservoir. Neutrophil chemotaxis was measured after 120 min at 37 °C, followed by image acquisition of the compartment below the transwell inserts. Each dot represents an independent experiment and line shows the mean. N = 6 different heifers. * P < 0.05, ** P < 0.01, *** P < 0.001 and **** P < 0.0001 compared to the control.

We first evaluated whether treatment with different BHB concentrations (0.5, 1.2, 1.4, 2.4, 3 and 10 mM) affected the chemotactic response of isolated bovine neutrophils using transwell assays. We observed a significant increase in bovine neutrophils chemotaxis at concentration ≥ 1.2 mM BHB (Fig. 1A,B). PAF was used as a positive control and induced a significant increase in neutrophil chemotaxis (Fig. 1C).

### Expression of the HCA2 receptor in bovine neutrophils

**Figure 2:**
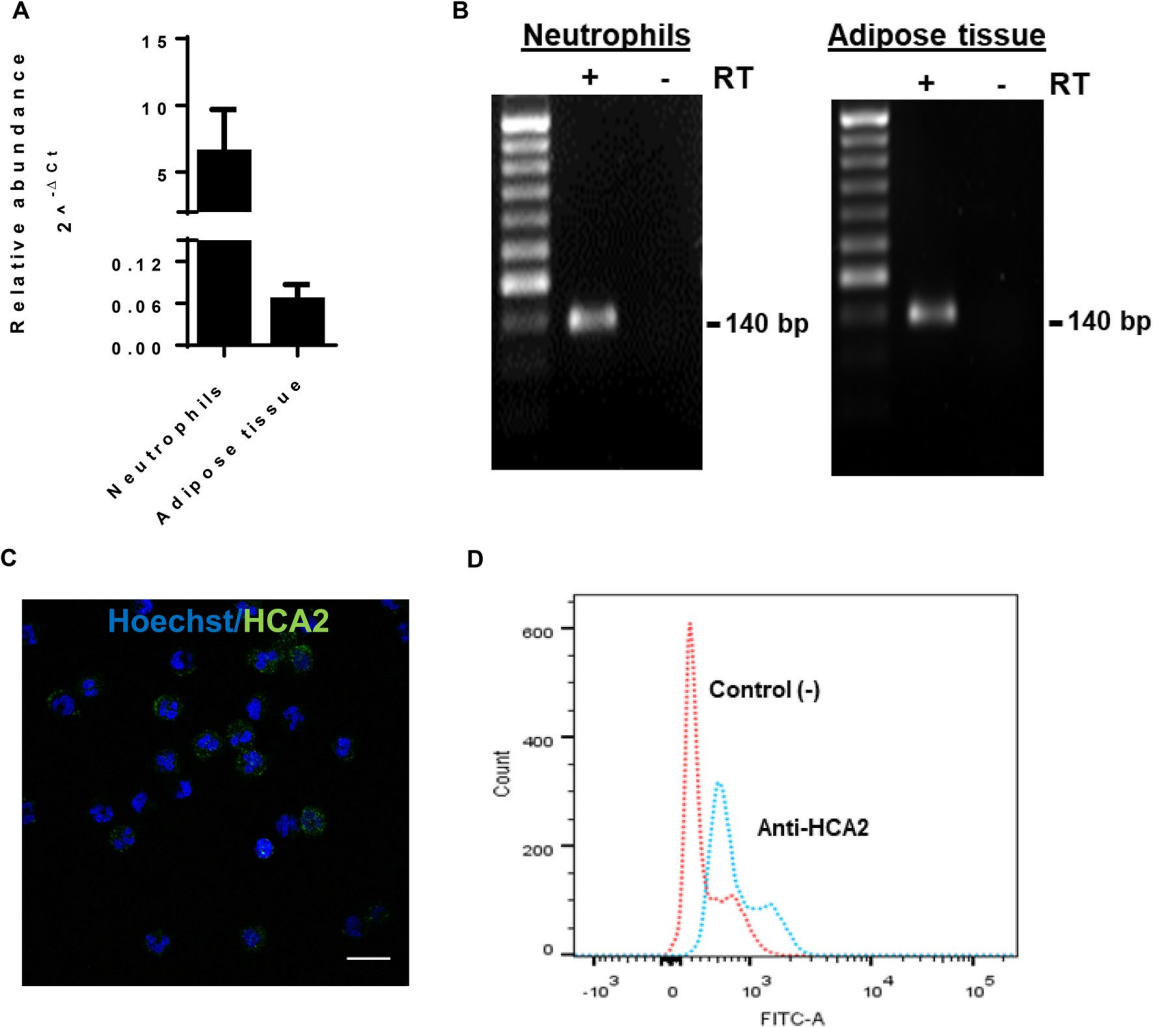
Analysis of HCA2 receptor expression in bovine neutrophils. (**A**) The expression of HCA2 mRNA in bovine neutrophils and adipose tissue was analyzed by real-time PCR. To exclude amplification of genomic DNA, RNA from cells was treated with DNAse before reverse transcription (RT +) or without reverse transcriptase (RT−). The data are expressed as the mean ± SEM of 9 samples from heifers or 3 samples for adipose tissue. (**B**) The size of the PCR products was checked by separation on 2% gels. A molecular size marker of 50 bp was used in the left lane. The product size was 140 bp. (**C**) Immunofluorescence for HCA2. Neutrophils were incubated with or without anti-HCA2 polyclonal antibody and Alexa 488-conjugated secondary antibody. The nuclei were dyed with Hoechst. The cells were analyzed by confocal microscopy. Bar = 25 µM. (**D**) Flow cytometry for HCA2 in bovine neutrophils. The cells were incubated with an anti-HCA2 antibody and Alexa 488-conjugated secondary antibody and analyzed with a FACSCanto II flow cytometer.

We evaluated expression of the HCA2 receptor in bovine neutrophils and kidney adipose tissue used as a positive control by real-time PCR using specific primers for HCA2. Figure 2A shows that the expression of HCA2 in neutrophils was higher than that in kidney adipose tissue. The size of the 140 bp PCR product in neutrophils and adipose tissue was confirmed by agarose gel electrophoresis (Fig. 2B). In addition, immunocytochemistry was performed and showed that HCA2 was primarily localized to the membrane (Fig. 2C). To corroborate receptor expression, neutrophils were incubated with an anti-HCA2 antibody and analyzed by flow cytometry. Figure 2D shows that an increase in fluorescence intensity on the X-axis was observed compared to that of the negative control containing only the secondary antibody.

### HCA2 agonists induce bovine neutrophil chemotaxis

**Figure 3:**
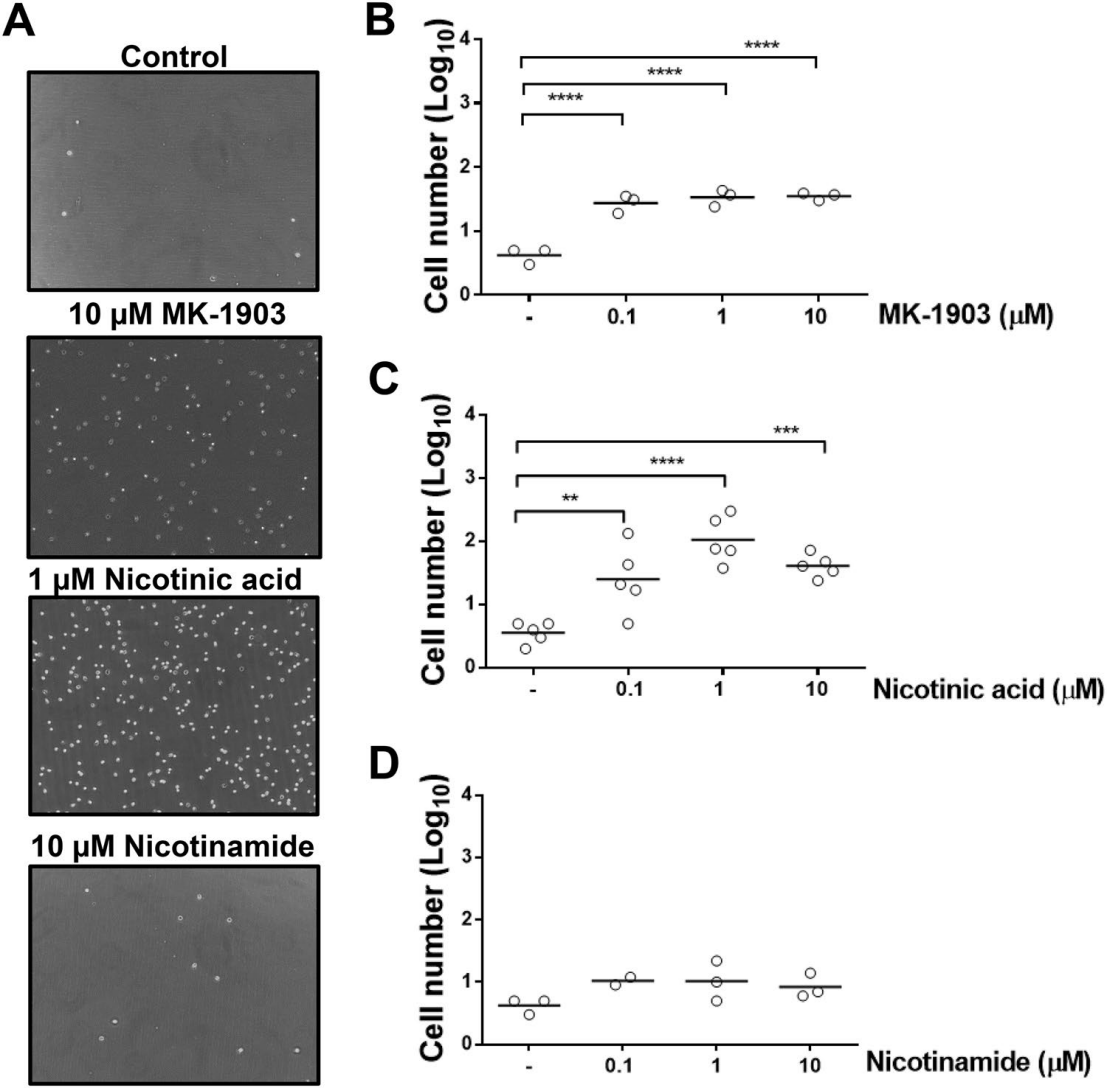
HCA2 receptor agonists increase bovine neutrophil chemotaxis. (**A**) Representative figures of neutrophil chemotaxis. A total of 1 × 10^6^ neutrophils were added to the upper part of the transmigration chamber and (**B**) MK-1903, (**C**) nicotinic acid and (**D**) nicotinamide were added to the lower reservoir. Neutrophil chemotaxis was measured after 120 min at 37 °C, followed by image acquisition of the compartment below the transwell inserts. Each dot represents an independent experiment and the line shows the mean. N = 3 different heifers. ** P < 0.01, *** P < 0.001 and **** P < 0.0001, compared to the control.

The chemotactic capacity of the different HCA2 receptor agonists was evaluated by transwell assays (Fig. 3A). MK-1903 (Fig. 3B) and nicotinic acid (Fig. 3C) induced a strong chemotactic response in neutrophils. As a control, we used a molecule that is structurally similar to nicotinic acid called nicotinamide that is not capable of activating the HCA2 receptor. We observed that nicotinamide did not induce a chemotactic effect in bovine neutrophils (Fig. 3D).

### HCA2 agonists induce calcium mobilization in bovine neutrophils

**Figure 4:**
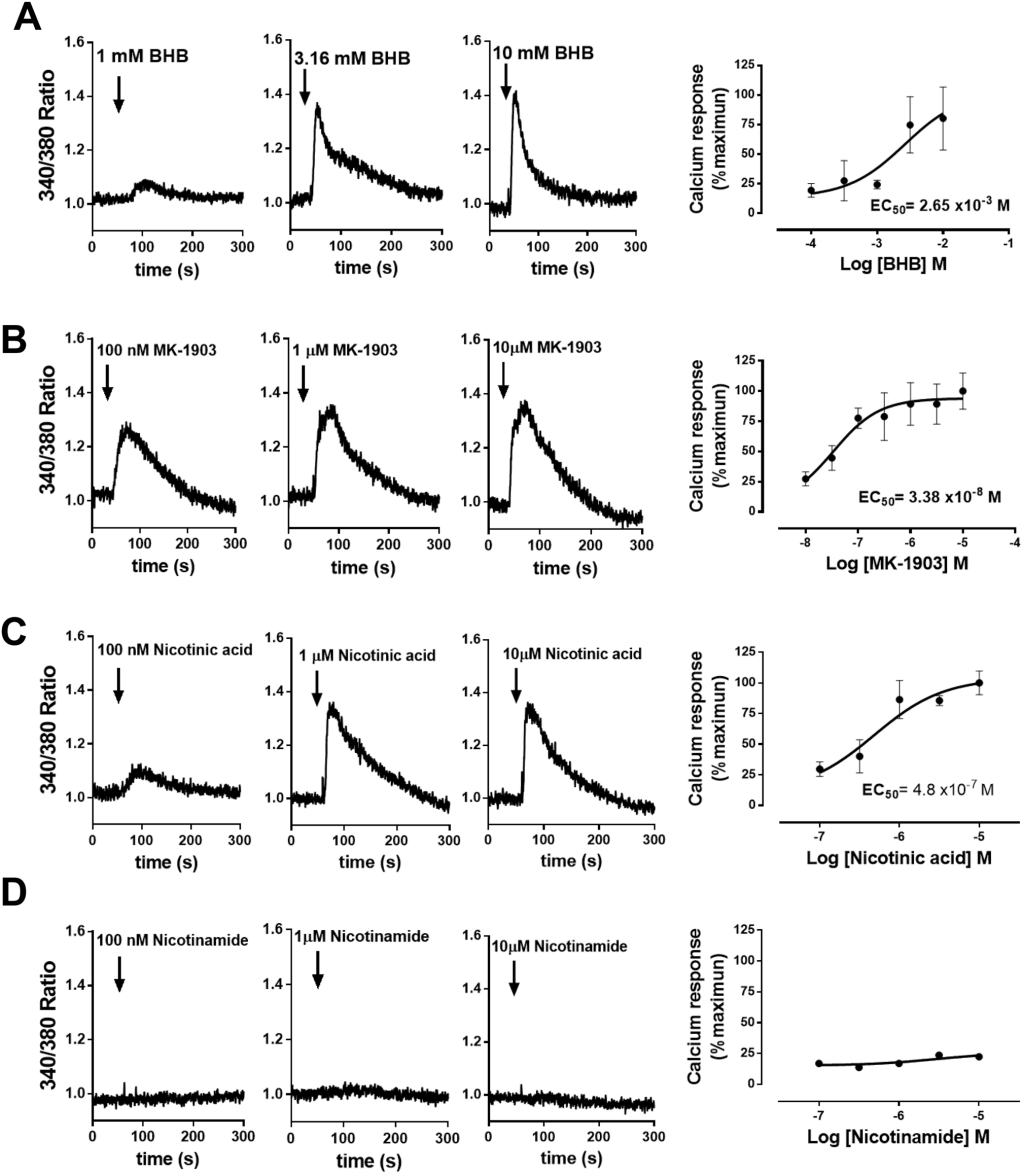
HCA2 receptor agonists induce calcium mobilization in bovine neutrophils. (**A**) Representative figures of the measured fluorescence of neutrophils labeled with Fura-2/AM probe and stimulated with (**A**) β-hydroxybutyrate (BHB), (**B**) MK-1903, (**C**) nicotinic acid and (**D**) nicotinamide. The arrows indicate the time at which the agonist was added. On the right are the dose response curves of HCA2 agonist-induced calcium influx in bovine neutrophils. The EC_50_ was calculated with GraphPad Prism v5.0 software.

To evaluate the effect of the different HCA2 receptor agonists on calcium mobilization in bovine neutrophils, these cells were labeled with the fluorescent Ca^2+^ probe Fura-2/AM. Figure 4 shows representative measurements of three different concentrations of agonists and the dose response curves of Ca^2+^ mobilization. A rapid increase in cytosolic Ca^ 2+^ was observed when the cells were stimulated with BHB (Fig. 4A), MK-1903 (Fig. 4B) and nicotinic acid (Fig. 4C), and this effect was dependent on concentration. Nicotinamide did not induce the mobilization of Ca^2+^ (Fig. 4D). We also calculated the EC_50_ by HCA2 agonists, and determined EC_50_ values of 2.65 mM, 33.8 nM and 480 nM for β-hydroxybutyrate, MK-1903 and nicotinic acid, respectively.

### HCA2 agonists activate different signaling pathways in bovine neutrophils

**Figure 5:**
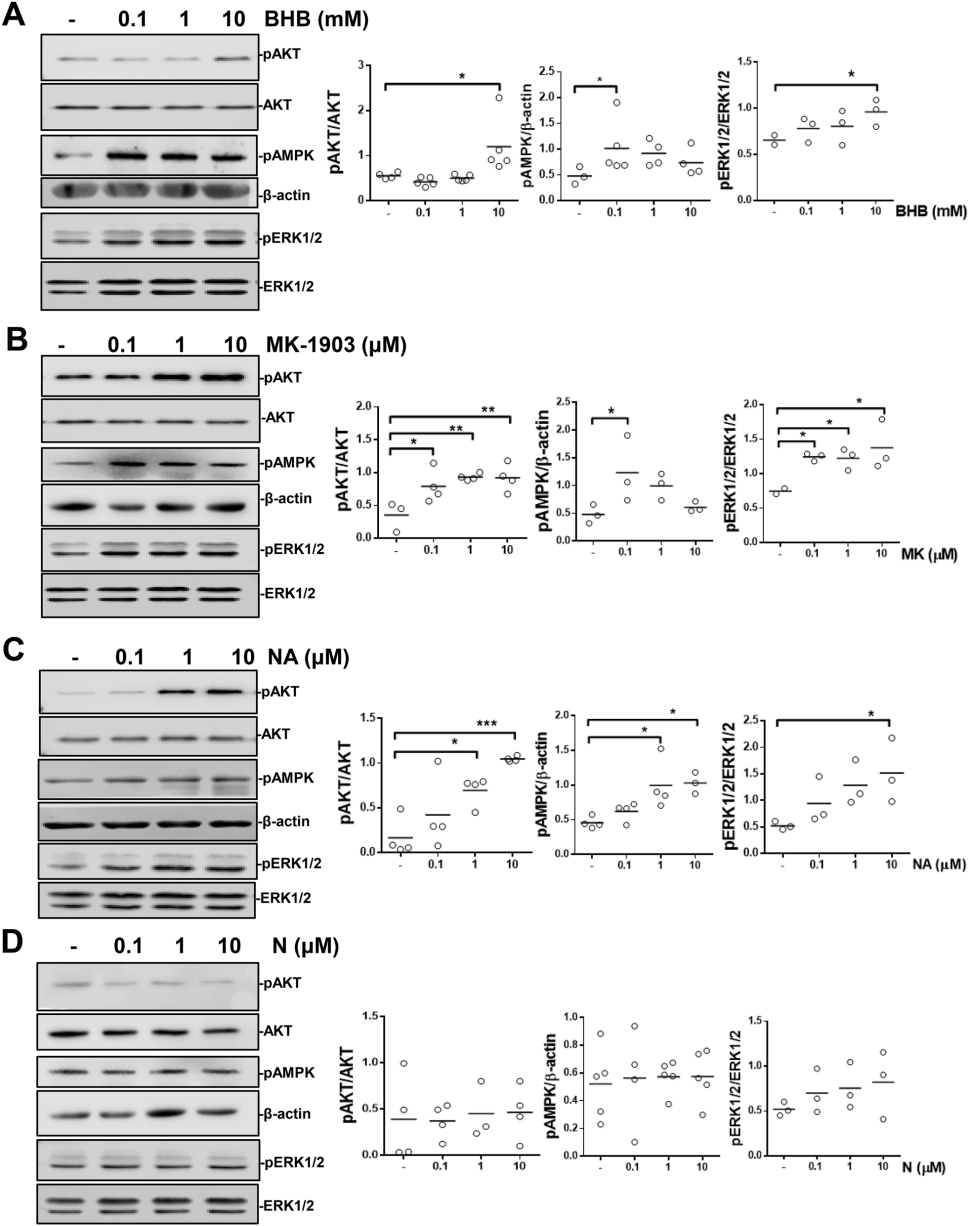
HCA2 receptor agonists increase AKT, AMPKα and ERK1/2 phosphorylation in bovine neutrophils. Neutrophils were treated for 3 min with (**A**) BHB, (**B**) MK-1903, (**C**) nicotinic acid and (**D**) nicotinamide. Total protein was analyzed by SDS/PAGE and immunoblotting using antibodies against the phosphorylated forms of AKT, AMPKα and ERK1/2. Unphosphorylated AKT, ERK 1/2 and β-actin were also analyzed, and the ratio was calculated by densitometric analysis. Each dot represents an independent experiment and the line shows the mean. N = 3 different heifers. * P < 0.05, ** P < 0.01, and *** P < 0.001 compared with the control.

We next determined whether the different HCA2 receptor agonists affect signaling pathways such as AKT, AMPK and ERK1/2. We found that 10 mM BHB significantly activated the AKT and ERK1/2 pathways. Additionally, 0.1 mM BHB induced AMPK activation (Fig. 5A). Similarly, all concentrations of MK-1903 activated the AKT and ERK1/2 pathways, and 0.1 µM MK-1903 induced AMPK activation (Fig. 5B). Nicotinic acid activated the three pathways in a concentration-dependent manner (Fig. 5C). In contrast, nicotinamide had no effect on these pathways (Fig. 5D).

### Involvement of Ca^2+^, PLC and Gi protein in HCA2-mediated chemotaxis

**Figure 6:**
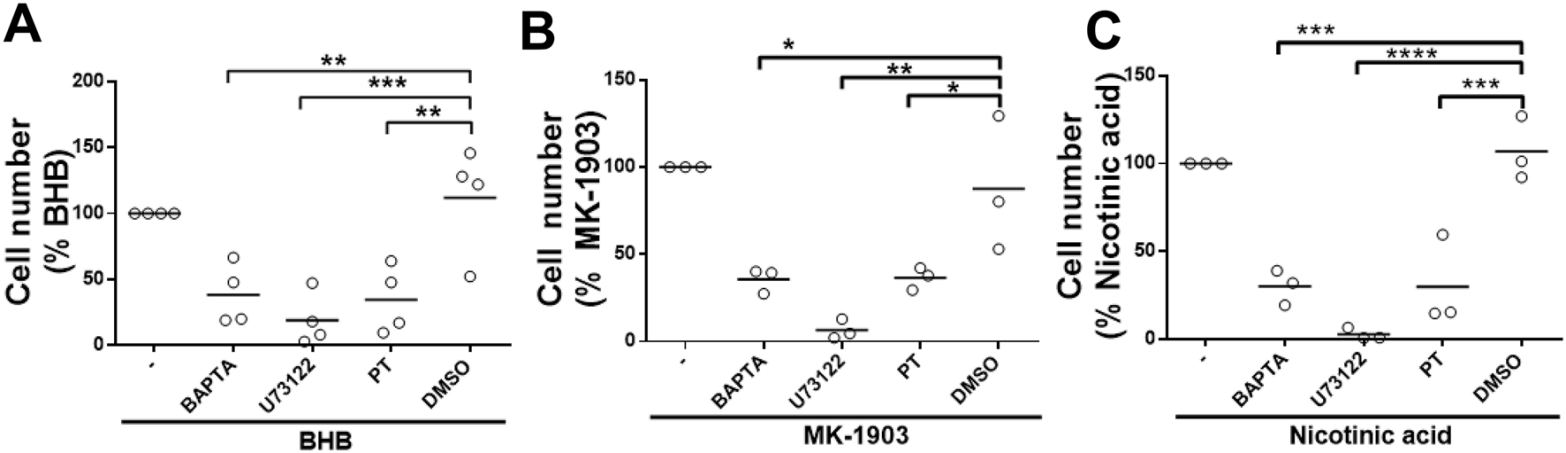
Role of Ca^2+^ signaling, Gi protein and PLC in HCA2 receptor agonist-induced chemotaxis. A total of 1 × 10^6^ neutrophils were incubated with the pharmacological inhibitors BAPTA-AM, U73122, and pertussis toxin for 30 min and then added to the upper reservoir. The HCA2 agonists (**A**) 10 mM BHB, (**B**) 10 µM MK-1903 and (**C**) 1 µM nicotinic acid were placed in the lower reservoir of the transmigration chamber. In all experiments, chemotaxis was determined after the neutrophils were allowed to migrate for 120 min at 37 °C, followed by imaging the cells in the lower reservoir. Each dot represents an independent experiment and the line shows the mean. N = 3 different heifers. * *P* < 0.05, **P < 0.01, ***P < 0.001 and ****P < 0.0001 compared with the DMSO treatment.

To explore the role of Ca^2+^ and PLC in the regulation of neutrophil chemotaxis induced by HCA2 agonists, transwell assays were performed by incubating the cells with the intracellular Ca^2+^ chelator BAPTA-AM and the non-selective PLC inhibitor U73122. Additionally, to evaluate whether the pertussis toxin-sensitive G_i_ subfamily of G proteins was involved in the regulation of chemotaxis, cells were incubated with 200 ng/mL pertussis toxin for 1 h. We use concentrations of the HCA2 agonists that elicit the main chemotaxis responses. Chemotaxis induced by 10 mM BHB was inhibited by U73122, BAPTA-AM and pertussis toxin (Fig. 6A). Dimethylsulfoxide (DMSO), the vehicle of the inhibitors, did not alter agonist-induced chemotaxis. The same response occurred with chemotaxis induced by 10 μM MK-1903 (Fig. 6B) and 1 μM nicotinic acid (Fig. 6C).

### AKT and ERK1/2 are involved in HCA2 agonist-induced chemotaxis

**Figure 7:**
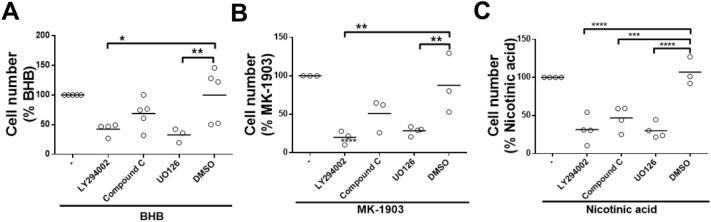
Role of AKT, AMPK and ERK1/2 activation in HCA2 receptor agonist-induced chemotaxis. A total of 1 × 10^6^ cells were incubated with the pharmacological inhibitors LY294002, compound C, and U0126 for 10 min and then added to the upper reservoir. The HCA2 agonists (**A**) 10 mM BHB, (**B**) 10 µM MK-1903 and (**C**) 1 µM nicotinic acid were placed in the lower reservoir of the transmigration chamber. In all experiments, chemotaxis was determined after the neutrophils were allowed to migrate for 120 min at 37 °C, followed by imaging the cells in the lower reservoir. Each dot represents an independent experiment and the line shows the mean. N = 3 different heifers. *P < 0.05, **P < 0.01, ***P < 0.001 and ****P < 0.0001 compared with the DMSO treatment.

To investigate whether the signaling pathways stimulated by HCA2 agonists are involved in chemotaxis in bovine neutrophils, cells were pretreated with 50 µM LY294002, 10 µM compound C and 10 µM UO126 to assess inhibition of the AKT, AMPK or ERK1/2 pathway, respectively. We observed that chemotaxis induced by 10 mM BHB and 10 µM MK-1903 was inhibited only with LY294002 and UO126 (Fig. 7A,B). The AMPK inhibitor did not have an effect on this response. However, chemotaxis induced by nicotinic acid was inhibited by all three inhibitors (Fig. 7C).

## Discussion

Our study clearly showed that concentrations of BHB observed in animals with ketosis such as 1.2 and 1.4 mM can induce bovine neutrophil chemotaxis in an in vitro transwell assay. 10 mM BHB induced the maximum response in neutrophils chemotaxis, but it is important to note that this concentration is non-physiological and should be carefully considered. Another important aspect to note is that our experiments were done with neutrophils from heifers, which respond very differently when compared with periparturient cows due to metabolic changes in this period^27,28^. For example, the chemotactic response of neutrophils was found to be significantly reduced in cows during the immediate post-partum period, in contrast to that of any other period^29^. This discrepancy may also be due to other factors that are impaired during postpartum such as elevated blood levels of NEFA^30^ , hypocalcemia^31,32^ and low glucose^33^. Other authors showed that neutrophils isolated from postpartum cows with ketosis or in an environment with ketone bodies are less capable of chemotaxis^34^. This difference can also may be due to the techniques used to evaluate chemotaxis. In our work, we used transwell assays that facilitate the analysis of the chemotactic effects of chemoattractants in comparison with the agarose technique in which cells are incubated in static conditions^35^. It has also been reported that there is significant within-day and between-day variation in the in vitro agarose chemotaxis assay in bovines^36^, which can underestimate chemotaxis levels. Additionally, another study showed that the migration of milk neutrophils from cows with clinical ketosis toward a chemoattractant was diminished^37^; however, it is important to consider the clear differences between milk and blood neutrophils. Structurally, milk neutrophils are smooth and spherical due to the loss of pseudopods caused by internalization of membrane material and contain numerous milk fat globules^38^. These morphological differences can influence the capacity of neutrophil chemotaxis.

In addition, our findings demonstrate that nicotinic acid and MK-1903 mimicked the effect of BHB in inducing bovine neutrophil chemotaxis. Other authors have shown in mouse neutrophils that dimethyl fumarate, a known HCA2 agonist, inhibits neutrophil chemotaxis toward the chemokine CXCL2 in transmigration assays^39^. In other immune cells, nicotinic acid inhibits macrophage chemotaxis in response to the chemoattractants fMLF and CCL2^40^. We observed that HCA2 agonists enhanced the chemotaxis induced by other chemoattractants, such PAF (Supplementary Fig. 1). These results suggest that HCA2 agonists have different functions in cattle compared with those of humans or other species. Other authors demonstrated that pharmacological amounts of nicotinic acid in cattle unexpectedly led to striking decreases in feed intake, which is a response that differs from that seen in nonruminants^41^. Additionally, other receptors, such as the bovine short fatty acid receptor FFA2, have different responses in comparison with those of humans and rodents^42^. For example, butyrate, a natural agonist of FFA2, has a proinflammatory effect in bovines^43^, rather than the antiinflammatory effect observed in human and mouse neutrophils^44^.

Currently, there are no known HCA2 receptor antagonists that could help to better understand the role of HCA2. Moreover, molecular techniques to silence gene expression are difficult because neutrophils are terminally differentiated cells that have a shorter lifespan than other immune cells^45^. Additionally, it has been demonstrated that the mere transfection of neutrophils with plasmid DNA itself represents a potent stimulatory condition for gene expression. These authors suggest that human neutrophils possess intracellular sensor systems that recognize foreign DNA and induce a potent immune response^46^ that may also be present in bovine neutrophils.

There are emerging data revealing the importance of the activation of HCA2 on inflammation in a number of tissues and clinical states in humans^47^. In bovines, the high metabolic demands caused by the use of intensive production systems lead to dramatic changes in the levels of BHB. For this, expression of the HCA2 receptor in the immune system and tissues involved in metabolic homeostasis in cattle supports a critical role of this receptor in the innate immune response in these animals^48^. Our results indicated that the expression level of HCA2 mRNA was higher in bovine neutrophils than in kidney adipose tissue. Consistent with this result, other authors have already demonstrated the presence of the HCA2 receptor in polymorphonuclear cells and adipose tissue^25,26,49^, but there were no data about the protein expression of the receptor in neutrophils. We confirmed that bovine neutrophils express the HCA2 receptor, as demonstrated by flow cytometry and immunofluorescence. Other authors localized the expression of this receptor in bovine fat and liver cells and related it with feed intake regulation and metabolism in cattle^25^.

In neutrophils, G_i_ protein activation mediates their effects via Gβγ subunits that have two intracellular effectors, the PLC β isoforms and γ-isoform of PI3K (PI3Kγ). PLC-β hydrolyzes PIP2 to produce inositol 1,4,5-trisphosphate (IP3), which stimulates intracellular calcium mobilization^50^. In this study, we observed that treatment with BHB and HCA2 agonists led to a rapid increase in calcium mobilization. Similar to this result, previous studies have also shown that activation of HCA2 by nicotinic acid induced a transient increase in calcium in human neutrophils^20^ as well as in macrophages^19^. Our results showed that BHB induces calcium mobilization with an EC_50_ value of 2.7 mM. Previous data have shown that BHB and butyrate activate human HCA2 with EC_50_ values of 0.7 mM and 1.5 mM, respectively^14^. There is some discrepancy in the role of intracellular calcium in chemotaxis. However, we observed a reduction in chemotaxis using the calcium chelator BAPTA-AM, indicating that this second messenger is necessary for chemotaxis induced by HCA2 agonists. Some authors have shown that inhibiting intracellular calcium with calcium chelating agents does not affect the chemotaxis induced by chemoattractants in the absence of adhesion molecules, but other studies showed that chemoattractant-induced chemotaxis of neutrophils on surfaces coated with adhesive molecules seems to depend on calcium release^51^.

Gi proteins are a class of G proteins that are sensitive to pertussis toxin. This bacterial toxin ribosylates Gαi/o, inactivating its function but not the function of the α subunits of the Gs, Gq or G12 classes^5^. We observed that pretreatment with pertussis toxin decreased the chemotactic response induced by HCA2 agonists, indicating that activation of Gi protein is essential for this process. Similar to this result, pertussis toxin has been shown to decrease the chemotactic response induced by fMLP and LBT4^52^.

Activation of the Gi protein-coupled receptor leads to the activation of PLC isoforms, but its role in neutrophil chemotaxis is still not well understood. There is evidence that both phospholipase C isoforms β2 and β3 are critical in chemoattractant-induced responses in murine neutrophils^51^. On the other hand, other authors revealed that PLCβ2 and PLCβ3 have an important role in the chemoattractant-mediated production of superoxide but not chemotaxis^53^. However, we observed that the PLC inhibitor U73122 strongly inhibited neutrophil chemotaxis induced by HCA2 agonists. Consistent with this result, a recent study has shown that treatment with U73122 reduces the chemotaxis of human neutrophils, suggesting an essential role of PLC signaling in neutrophil chemotaxis^54^.

PI3Kγ exhibits protein kinase activity, and its major target is serine-threonine protein kinase B (PKB/Akt), which is required for chemotaxis^55^. Our results clearly showed that all HCA2 agonists induced rapid phosphorylation of AKT within 3 min. Other authors have also shown that nicotinic acid evoked significant Akt phosphorylation in a time-dependent fashion, with maximal activation at 5 min in CHO-HCA2^56^ and RAW264.7 cells^40^. It is well known that fasting or starvation increases ketone body metabolism in the brain, and this is related to an increase in phosphorylation of AKT in the prefrontal cortex of mice^57^. In dairy cattle, recent studies have shown that skeletal muscle changes due to parturition and lactation with hyperketonemia are related to the phosphorylation status of AKT^58^. Additionally, our study showed that inhibition of this pathway with LY294002 decreased the chemotactic response elicited by all HCA2 agonists. Consistent with these results, other studies have shown that using LY294002 and wortmannin decreased the migration of neutrophils in response to IL-8 and LTB4^59^.

Almost all GPCRs signal through MAPK cascades, which are usually associated with control of cell proliferation, differentiation, apoptosis and directed migration of leukocytes^60,61^. In this study, we found that HCA2 agonists significantly stimulated MAPK ERK1/2 activation in a concentration-dependent fashion. Previous studies showed that treatment with nicotinic acid for 5 min elicits activation of ERK1/2 in RAW264.7 cells^40^. Other authors demonstrated that nicotinic acid and other flushing compounds, such as 5-methyl-3-carboxyl-pyrazole, induced activation of ERK1/2 in CHO-k1 cells^62^. Our results are consistent with rapid stimulation of the intracellular pathway mediated by GPCR activation. We also observed that this pathway is involved in the chemotactic response elicited by HCA2 agonists because pretreatment of neutrophils with UO126 resulted in a significant decrease in chemotaxis. However, another study showed that UO126 reduced nicotinic acid-mediated inhibition of mouse macrophage chemotaxis in response to chemoattractants such as fMLP^40^. This discrepancy may be due to the different responses of this receptor in bovines.

AMPK is a heterotrimeric complex that is involved in the regulation of energy metabolism and is activated under conditions that deplete cellular ATP and elevate AMP levels, such as those that occur during glucose deprivation and hypoxia. In bovine ketosis, in addition to increased serum BHB, a decrease in serum glucose concentrations has also been reported^63^. Moreover, it has been demonstrated that BHB activates the AMPK signaling pathway and regulates lipid synthesis in bovine hepatocytes^64^. Our present study showed that all HCA2 agonists induced AMPK activation in bovine neutrophils. Other authors also demonstrated that nicotinic acid stimulates the expression of AMPK in bovine adipocytes^65^ and that BHB increases AMPK activity in dairy cow anterior pituitary cells^66^. In the present study, inhibition of AMPK did not interfere with the chemotaxis induced by 10 mM BHB or 10 μM MK-1903 in neutrophils. This confirms that the AMPK pathway is only activated at low concentrations of these agonists, which are not related to the chemotaxis response. In contrast, chemotaxis induced by 1 µM nicotinic acid induced AMPK phosphorylation and chemotaxis that was decreased by compound C, suggesting that this pathway is necessary for this event. These results are consistent with other studies that demonstrated that AMPK activation is required for mouse neutrophil chemotaxis^7^ and epithelial and T cell migration^67,68^.

Based on our data, we propose that BHB and the synthetic agonists MK-1903 and nicotinic acid activate the HCA2 receptor. Receptor activation causes the dissociation of Gi proteins from Gβγ subunits, triggering activation of PLC coupled to calcium release and phosphorylation of AKT, AMPKα and ERK1/2, which are pathways that are involved in HCA2 agonist-induced chemotaxis.

## Conclusions

Neutrophils isolated from heifer highly express the HCA2 receptor and their activation with the endogenous (BHB) and synthetic (MK-1903, Nicotinic acid) agonists increases bovine neutrophil chemotaxis. Also, these agonists led to rapid and transient changes in calcium levels that were concentration-dependent, indicating that bovine neutrophils express a functional HCA2 receptor. This second messenger as well as PLC and Gi protein are involved in HCA2-mediated chemotaxis. Phosphorylation of AKT, ERK 1/2 and AMPKα are also crucial for the neutrophil chemotaxis mediated by HCA2 activation. These observations may provide new insights into the pharmacological effects of and the physiological functions modulated by the β-hydroxybutyrate receptor HCA2 in neutrophils. However, more studies considering range of BHB concentrations as well as neutrophils from cows at different parturient state would be useful for a complete understanding of the mechanisms that control chemotaxis and the role of ketosis in livestock immunity.

## Methods

### Animals

Six clinically healthy non-pregnant and non-lactating Holstein Friesian heifers with body weights of 280–310 kg from the Austral University herd were used in all experiments. Heifers were maintained at a large animal farm on an ad libitum grass diet with grain supplementation. All experiments were conducted in strict accordance with the recommendations of the “Comisión Nacional de Investigación Científica y Tecnológica” and the current Chilean Animal Protection Laws by the ethics committee of the Universidad Austral de Chile. The protocol was approved by the ethics committee of the Universidad Austral de Chile (permit number: 270/2016).

### Isolation of neutrophils

Blood was collected aseptically by jugular vein puncture into acid citrate dextrose-lined collection tubes (Becton Dickinson, USA). Neutrophils were isolated according to a previously described method^69^. Viability was determined by trypan blue exclusion and was always at least 97%. Neutrophil purity was at least 94%, as assessed by flow cytometry (FACSCanto II, Becton Dickinson, USA), using a forward-scatter versus side-scatter dot plot to determine the relative size and granularity of the cells, which provides an effective method to assess the activation of cells^70^. Neutrophils were suspended in Hank’s Balanced Salt Solution (HBSS) and immediately used in the whole experiments.

### RNA isolation and RT-PCR analysis

Adipose tissue around the kidneys was aseptically removed from fetuses of cows that were found in a local abbatoir after slaughter, and immediately transferred to a tube with RNA later Stabilization Solution to process them later. Total RNA from 10 × 10^6^ neutrophils or 30 mg of adipose tissue was extracted using the EZNA total RNA Kit (E.Z.N.A., Promega, USA). Samples were treated with Turbo DNase-Free (Thermo Fisher Scientific, USA) to remove genomic DNA, and the RNA was quantified by using a NanoDrop 2000 spectrophotometer (Thermo Fisher Scientific, USA). We checked the quality of the RNA using the Agilent Fragment Analyzer 5,200 machine, with RIN values for neutrophils and adipose tissue of 6,4 and 8 respectively. For cDNA synthesis, 200 ng of total RNA was reverse transcribed in the presence or absence of reverse transcriptase by using Affinity Script QPCR cDNA synthesis kits (Agilent Technologies, USA). Real-time PCR assays were performed using Brilliant II SYBR Green QRT-PCR master mix (Stratagene, USA) and primers specific for bovine HCA2^25^. HCA2 mRNA levels were normalized using glyceraldehyde 3-phosphate dehydrogenase (GAPDH) with the forward primer 5′GGCGTGAACCACGAGAAGTATAA3′ and reverse primer 5′CCCTCCACGATGCCAAAGT3′. The following conditions were used in a qPCR StepOne system (Thermo Fisher, USA): 40 cycles at 95 °C for 60 s, 61 °C for 30 s (annealing), and 72 °C for 30 s (extension). The mRNA expression was calculated using the formula: relative abundance = 2^(−ΔCt)^, where ΔCt is the difference between the Ct (threshold cycle) of HCA2 and GAPDH. Products were separated on 2% agarose gels and stained with SYBR Safe DNA Gel Stain (Thermo Fisher, USA) for analysis.

### Immunocytochemistry

Neutrophils (1 × 10^6^) were placed in a slide (previously treated with 0.03% polylysine) with 4% paraformaldehyde for 20 min at 37 °C. The cells were washed with phosphate-buffered saline (PBS) and then incubated with a buffer according to the antibody manufacturer's instructions containing 0.1% Tween-20, 1% bovine serum albumin and 0.3 M glycine in PBS for 1 h. The slides were then incubated with rabbit polyclonal anti-Puma gamma/GPR109A antibody (1:100 dilution) (81,825 Abcam, USA) overnight. Finally, cells were then incubated with Alexa Fluor 488 goat anti-rabbit IgG secondary antibody (1:200 dilution) (Molecular Probes, USA). To visualize the nuclei, the slides were stained with Hoechst (1:4,000 dilution) (H3570 Invitrogen, USA) and then analyzed using a confocal microscope (FluoView FV1000, Olympus).

### Flow cytometry

Neutrophils (2 × 10^6^) were placed in flow cytometry tubes and centrifuged at 600 g for 5 min, and then 4% PFA was added for 10 min at RT. The cells were washed with 1 × PBS and permeabilized for 15 min with 0.5% Triton X-100. Then, the cells were washed and incubated with the primary anti-Puma gamma/GPR109A antibody (ab81825) (1:100 dilution) in blocking solution (1% BSA and 0.1% Tween in PBS 1 ×) at 4 °C overnight. The next day, the cells were incubated for two hours with a rabbit anti-IgG secondary antibody conjugated to Alexa Fluor (1:1,000 dilution) at RT. After removing excess antibody with PBS 1 × , the cells were resuspended in 300 µL of PBS 1 × and analyzed on a FACSCanto II cytometer. A negative control was prepared without primary antibody.

### Intracellular calcium measurements

Ca^2+^ influx was measured spectrofluorometrically using Fura-2-acetoxymethyl ester (Fura-2AM) (Molecular Probes, USA) as previously described^69^. First, a baseline fluorescence measurement was performed for 60 s before adding the agonists and then the recording was continued until 300 s were completed. The influx of calcium was measured as the increase in the 340/380 fluorescence ratio.

The dose–response curves of Ca^2+^ influx in the presence of the HCA2 agonists are expressed as a percentage of the maximum response and were analyzed using a nonlinear regression method, and the 50% effective concentration (EC_50_) was calculated with GraphPad Prism v5.0 software (GraphPad Software Inc.).

### Western blotting

Neutrophils (5 × 10^6^) were suspended in HBSS with 0.9 mM CaCl_2_ (HBSS + Ca^2+^) and incubated with 0.1, 1 and 10 µM nicotinic acid (Sigma–Aldrich, USA), MK-1903 (Tocris, Bristol, UK) or nicotinamide (Sigma–Aldrich, USA) and 0.1, 1 and 10 mM BHB (Sigma–Aldrich, USA) for 3 min at 37 °C. The cells were lysed and analyzed according to a previously described method^43^. Eighty micrograms of total protein was separated by 12% SDS-PAGE, transferred onto a nitrocellulose membrane and blocked in blocking solution [1 × Tris-buffered saline (TBS), 0.1% Tween-20 and 5% w/v nonfat dry milk] for 2 h at room temperature. The membranes were then incubated with the indicated primary antibodies overnight at 4 °C. Primary antibodies against phospho-ERK MAPK, phospho-AKT and phospho-AMPKα (Cell Signaling, USA) were used according to the instructions provided by the manufacturer. A secondary goat anti-rabbit antibody (1:10,000 dilution) (Licor, Lincoln, NE, USA) was used, and immunoreactivity was visualized using the SuperSignal West Femto maximum sensitivity substrate (Thermo Fisher, USA). Detection was performed on an Odyssey Fc Imaging System (LI-COR). The primary antibody was removed by incubation with a stripping solution (100 mM 2-mercaptoethanol; 2% SDS; 62.5 mM Tris–HCl, pH 6.7) for 1 h at 50 °C with agitation, followed by several washes with TBS-Tween 0.1%. The membrane was then incubated with a polyclonal ERK 1 or AKT antibody (Cell Signaling, USA) at a dilution of 1:2000, and β-actin HRP (Santa Cruz Biotechnology, USA) was also used. The densitometry of the protein bands was analyzed by using ImageJ 1.51j8 software.

### Chemotaxis assay

Chemotaxis was evaluated by transwell migration assay using 3-μm-pore transwell filters (Millipore, MA, USA) in a 24-well plate format. Nicotinic acid, MK-1903 and nicotinamide (0.1, 1 and 10 μM), BHB (0.1, 1 and 10 mM) or PAF (100 nM) was added to the bottom chamber of the transwell. A total of 1 × 10^6^ neutrophils in 500 μl of HBSS + Ca^2+^ were placed in the top chamber. After 2 h at 37 °C in 5% CO_2,_ the upper surface was removed, and the migrated cells on the lower surface were analyzed by light microscopy. Additionally, cells were incubated with 200 ng/mL pertussis toxin (Calbiochem, USA) for 1 h and 50 μM BAPTA-AM (Molecular Probes, USA) for 30 min, or 2 μM U73122 (Calbiochem, USA), 10 μM Compound C (Calbiochem, USA)^7^, 50 μM LY2903 (Promega, USA) according to manufacturer instructions and 10 μM UO126 (Promega, USA)^71^ for 10 min and then placed in the upper transmigration chamber, and 10 mM BHB, 1 μM nicotinic acid or 10 μM MK-1903 was placed in the lower reservoir.

### Statistical analysis

All assays are showed as scatter dot plot with mean or bar graphs of at least 3 independent experiments. Chemotaxis data were expressed as Log_10_. Our data showed normal distribution and variance homogeneity according to Shappiro-Wilks and Bartlett’s test respectively. For comparisons of two experimental groups, two-tailed unpaired t-test was used, while comparisons of three or more groups were performed using one-way analysis of variance (ANOVA) test followed by a Fisher’s LSD post hoc test. All statistical analyses were performed using GraphPad Prism v6.0 (GraphPad Software, La Jolla, CA, USA). Differences were considered significant at a P < 0.05 value.

Supplementary Fig. 1. HCA2 agonists enhanced the chemotactic response of PAF in bovine neutrophils. (A) A total of 1 × 10^6^ neutrophils were pretreated with HCA2 agonists for 1 h and then placed in the upper part of the transmigration chamber, and PAF was placed in the lower reservoir. In all experiments, chemotaxis was determined after the neutrophils were allowed to migrate for 120 min at 37 °C, followed by imaging the cells in the lower reservoir. (B) Each dot represents an independent experiment and the line shows the mean. N = 5 different heifers. < *P < 0.05 and ** P < 0.01 compared to the PAF alone.

## Supplementary information

Supplementary information.
