# Supplementary material for: β-hydroxybutyrate and hydroxycarboxylic acid receptor 2 agonists activate the AKT, ERK and AMPK pathways, which are involved in bovine neutrophil chemotaxis
Source: Sci Rep. 2020 Jul 27;10:12491. doi: 10.1038/s41598-020-69500-2 (PMC7385489; doi:10.1038/s41598-020-69500-2)

β-hydroxybutyrate and hydroxycarboxylic acid receptor 2 agonists activate the AKT, ERK and AMPK pathways, which are involved in bovine neutrophil chemotaxis

María D Carretta[1]*, Yonathan Barría[1], Katherine Borquez[1], Bárbara Urra[1], Andrés Rivera[1], Pablo Alarcón[1], María A Hidalgo[1], Rafael A Burgos[1].

1. Laboratory of Inflammation Pharmacology, Faculty of Veterinary Science, Institute of Pharmacology and Morphophysiology, Universidad Austral de Chile, Valdivia, Chile.

Contact corresponding author: daniellacarretta@gmail.com

Supplementary Figure 1

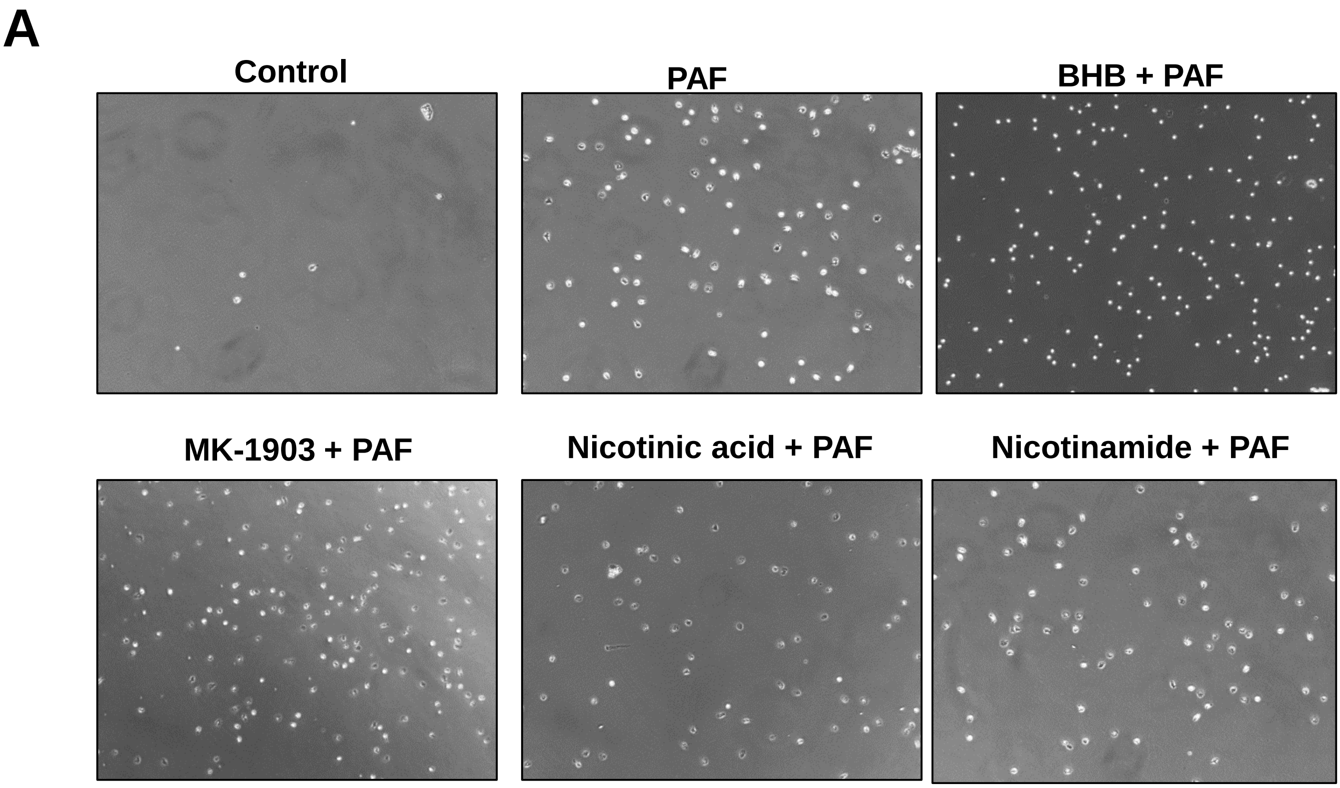

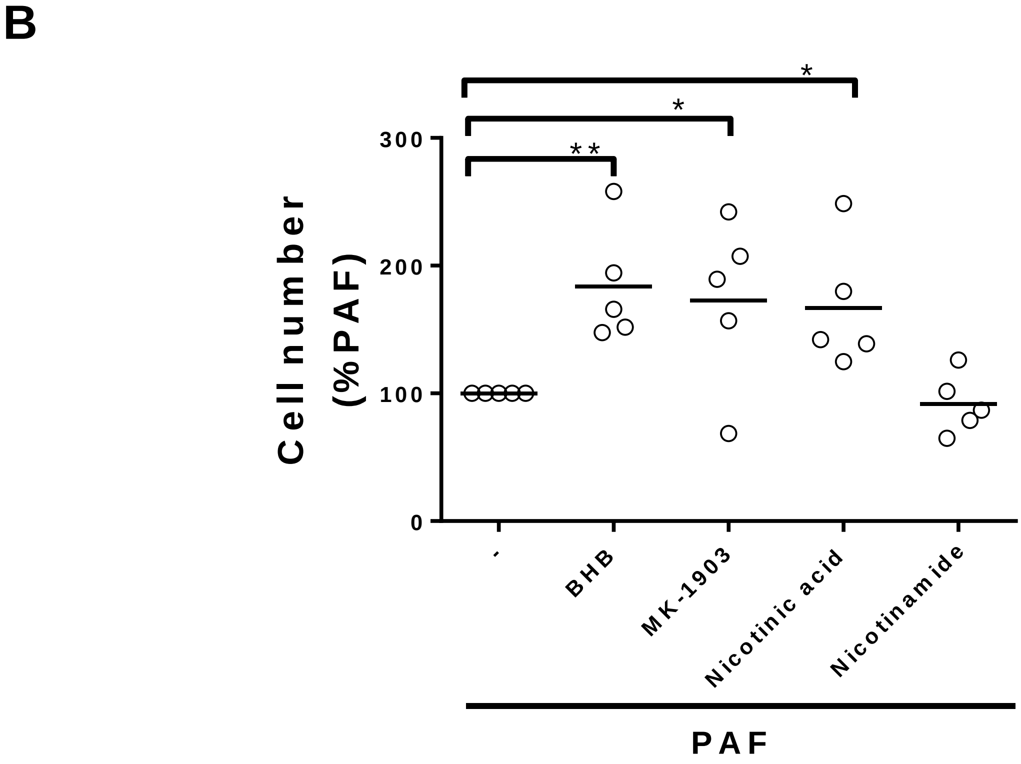

Supplementary Gel Images

We indicate the contours of the membranes by marked frames and the image which is shown in Figure 5

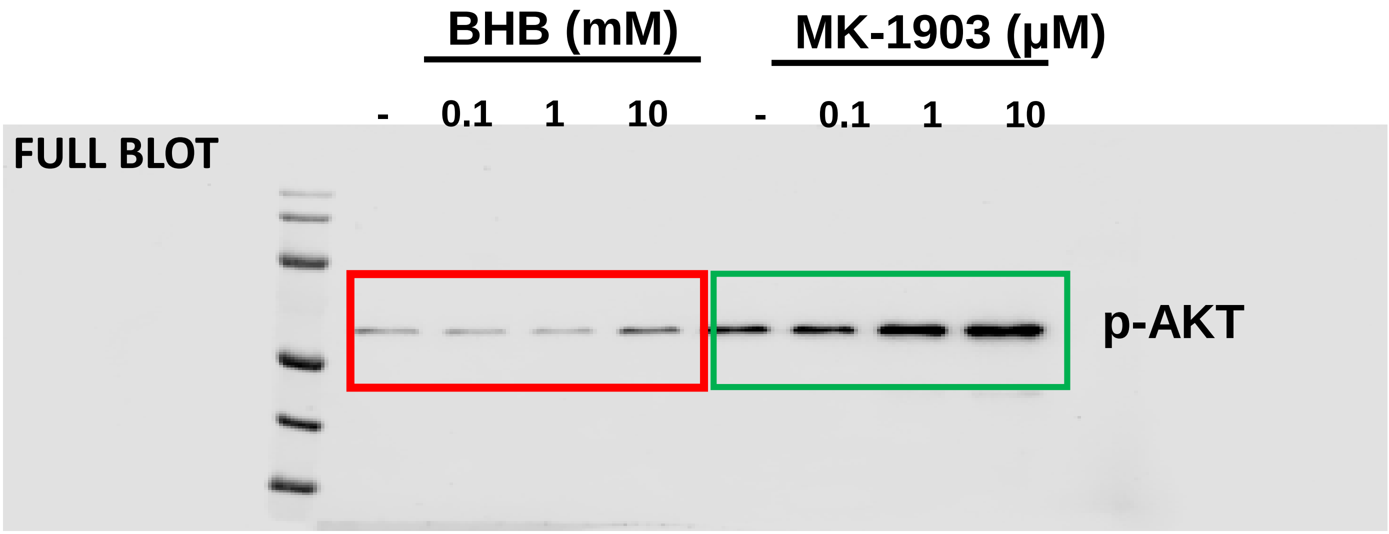

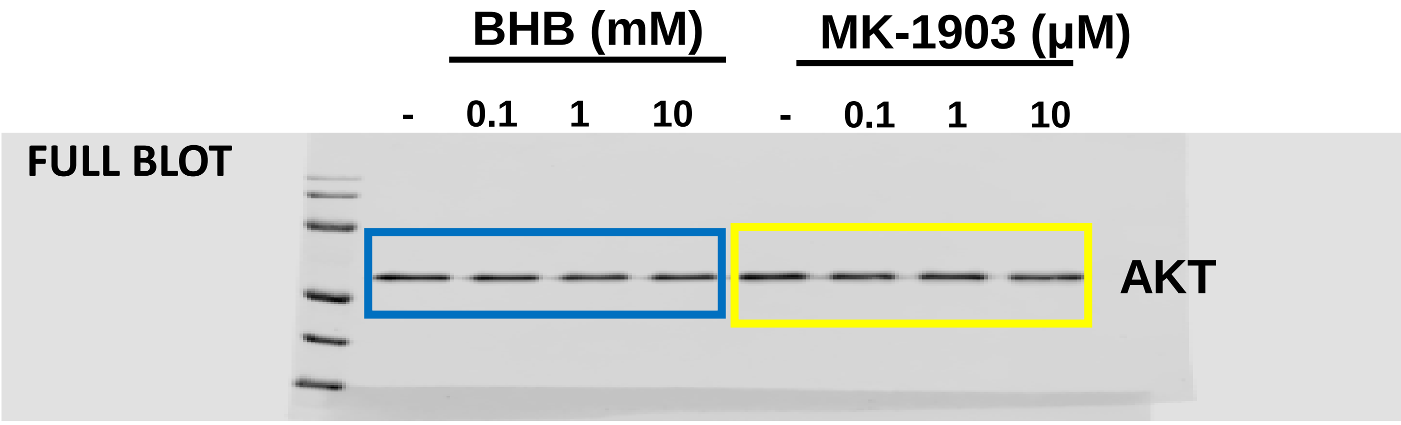

FIGURE 5 IN PAPER

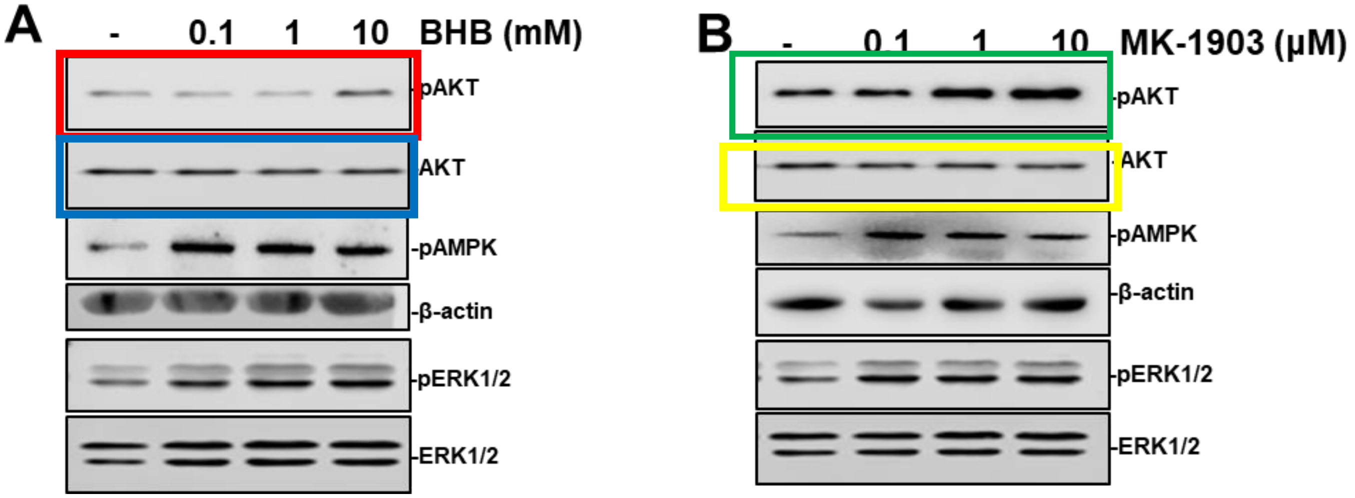

Supplementary Gel Images

FULL BLOT

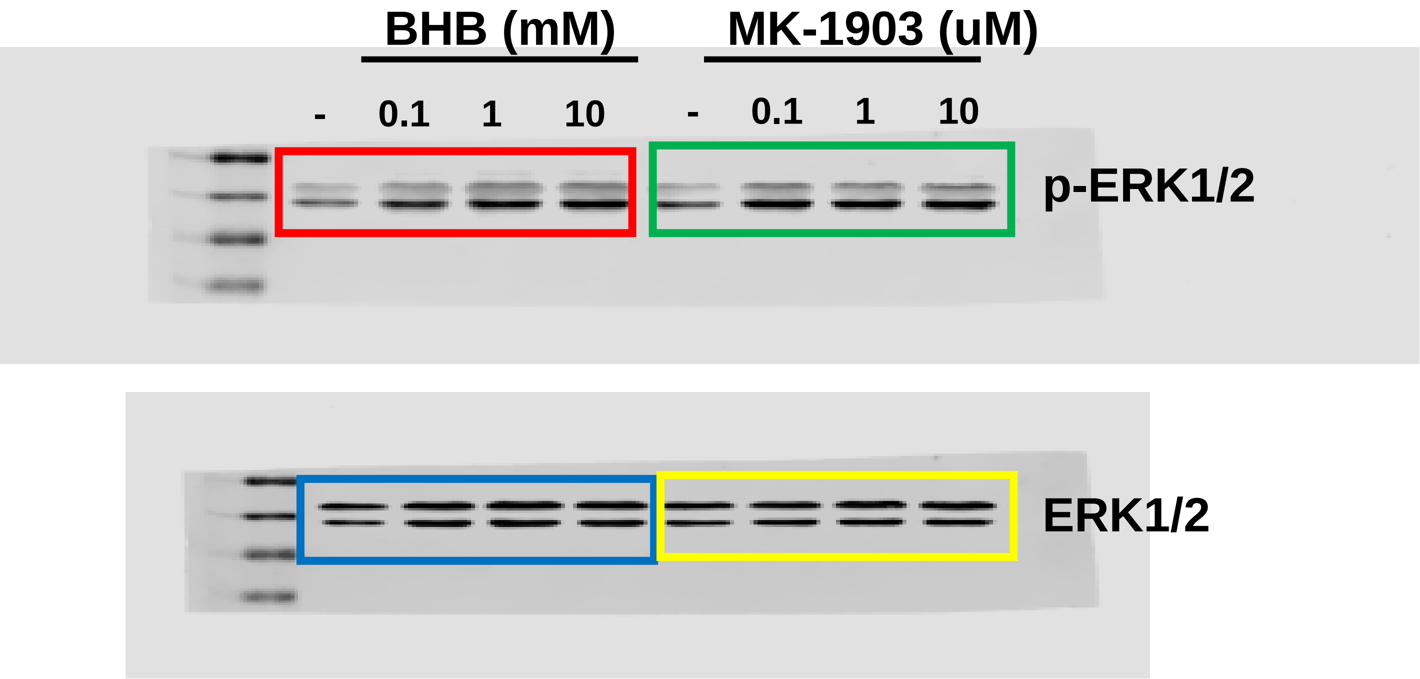

FIGURE 5 IN PAPER

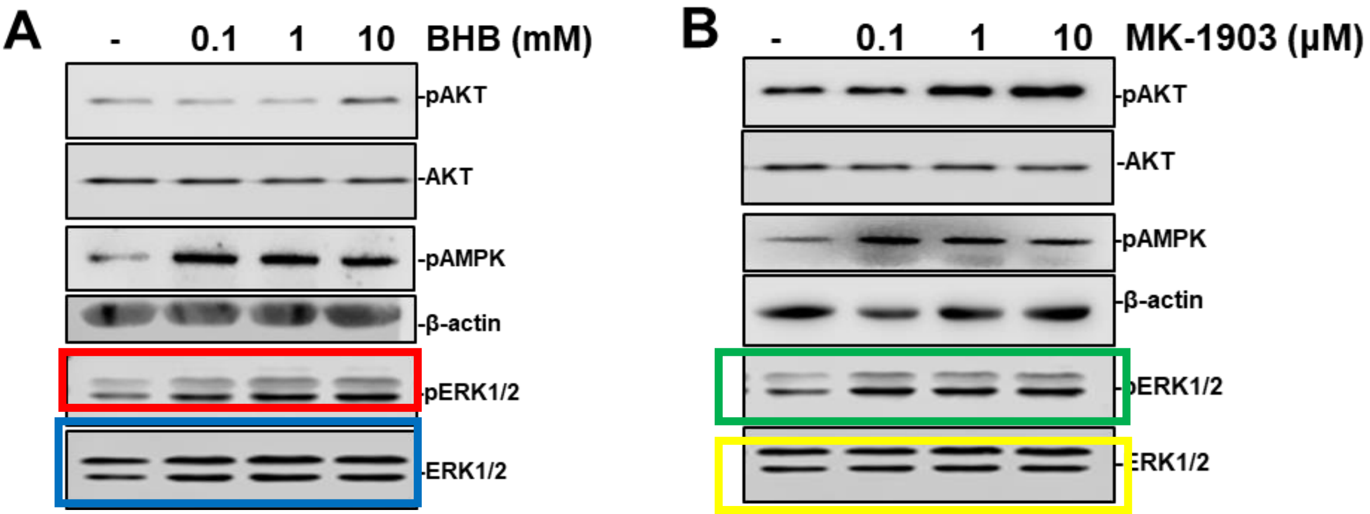

Supplementary Gel Images

FULL BLOT

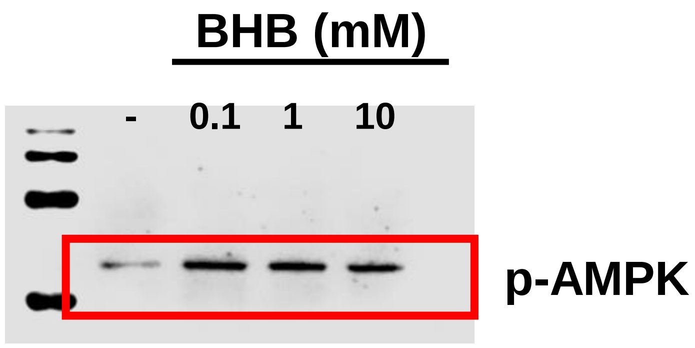

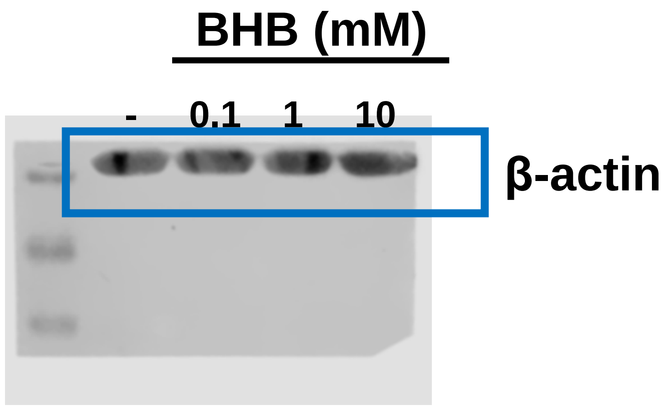

FIGURE 5 IN PAPER

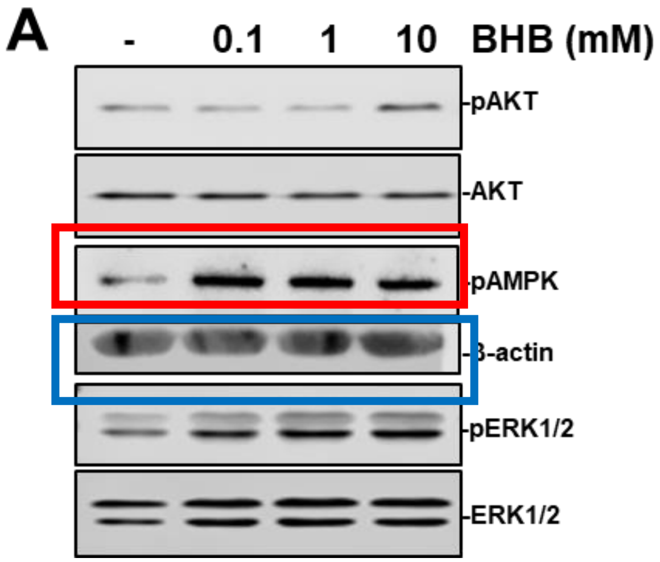

Supplementary Gel Images

FULL BLOT

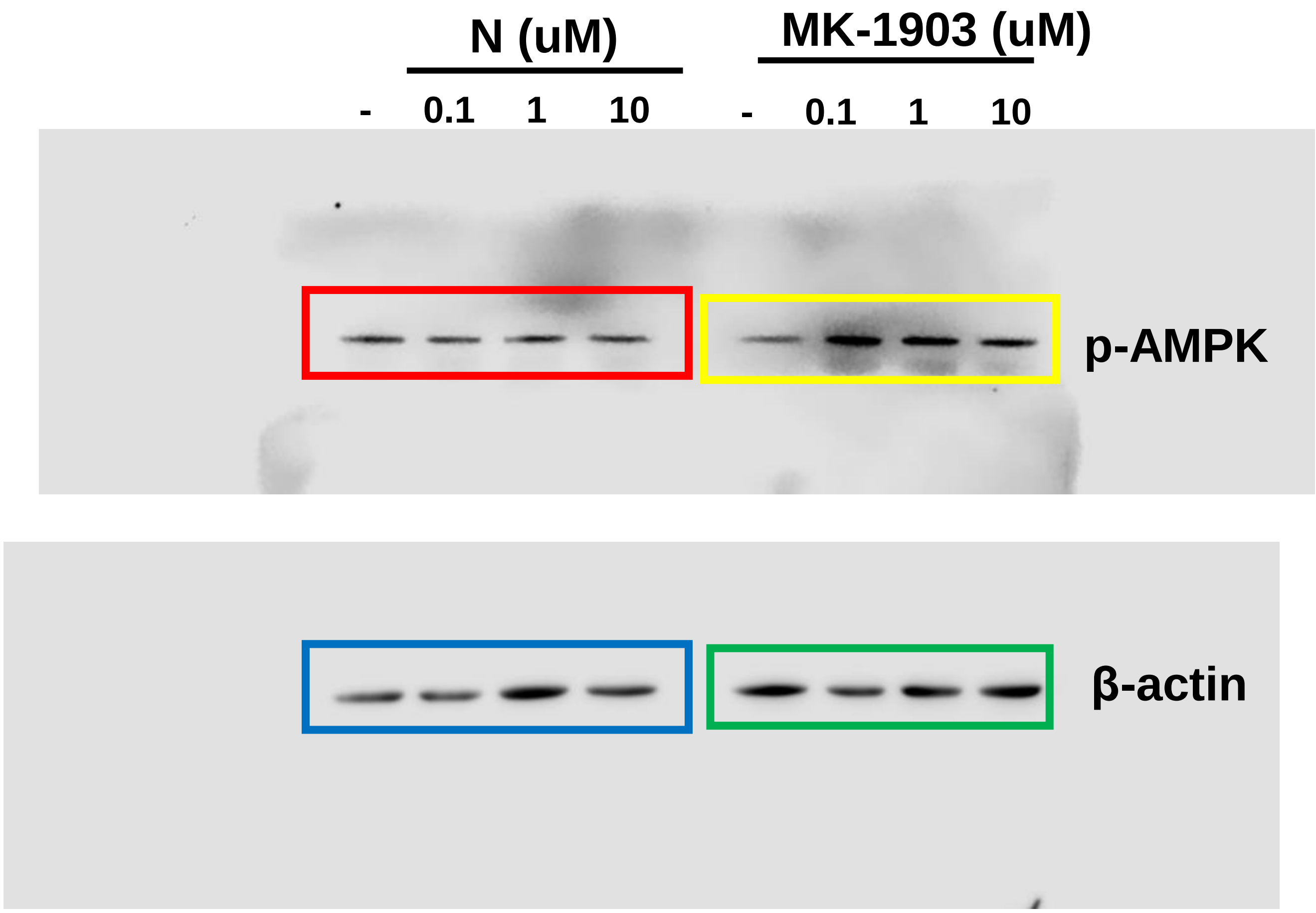

FIGURE 5 IN PAPER

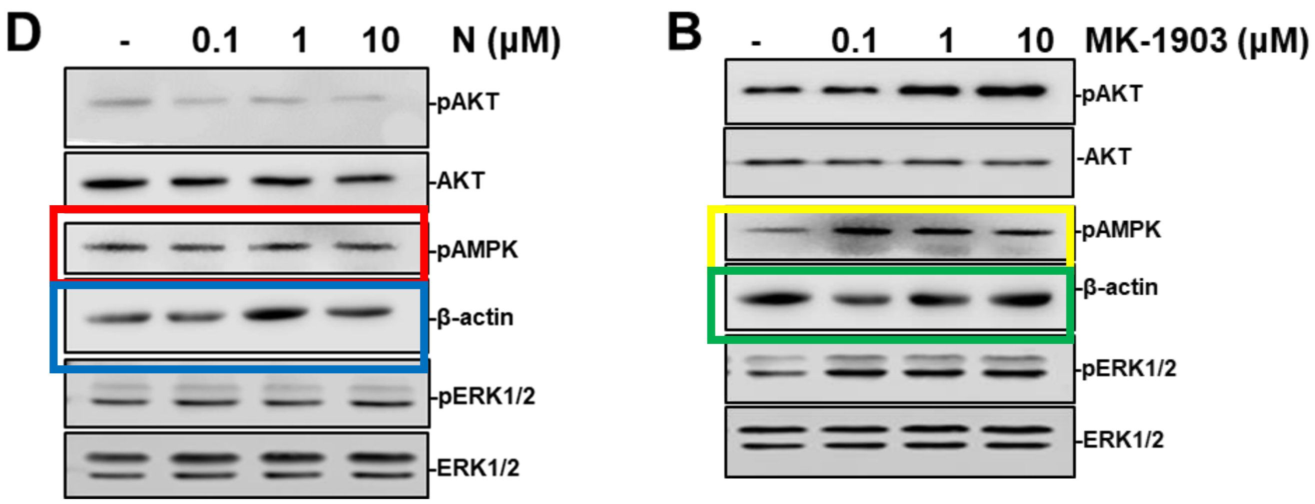

Supplementary Gel Images

FULL BLOT

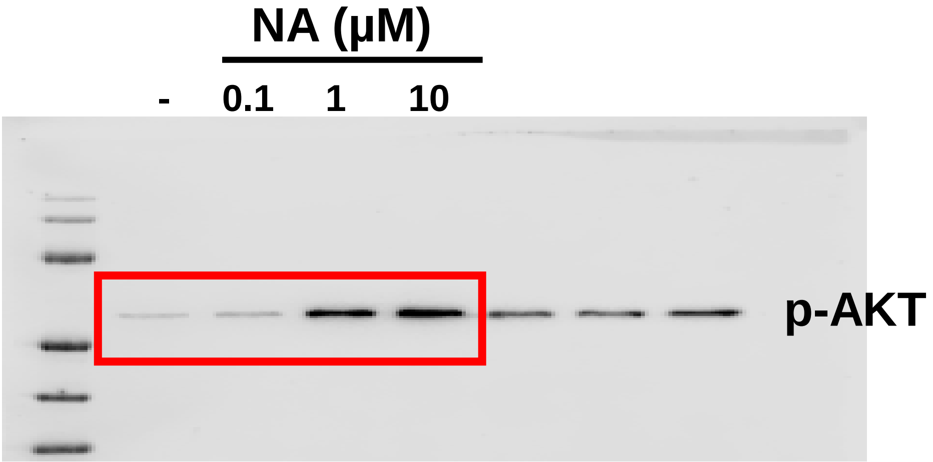

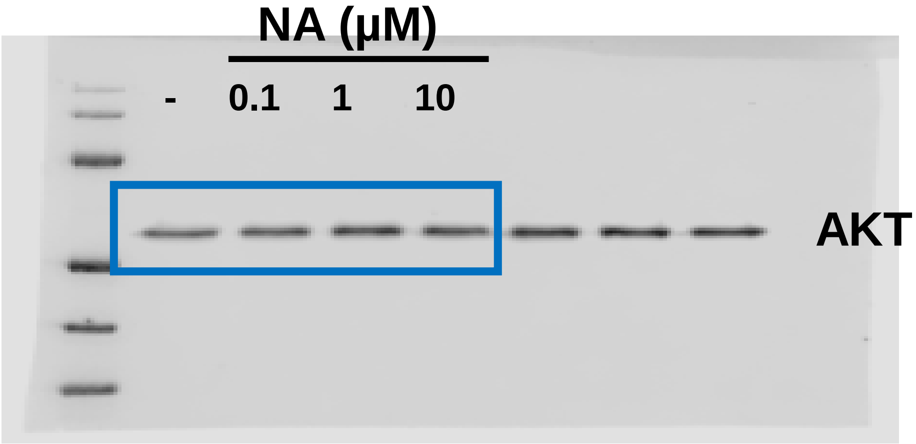

FIGURE 5 IN PAPER

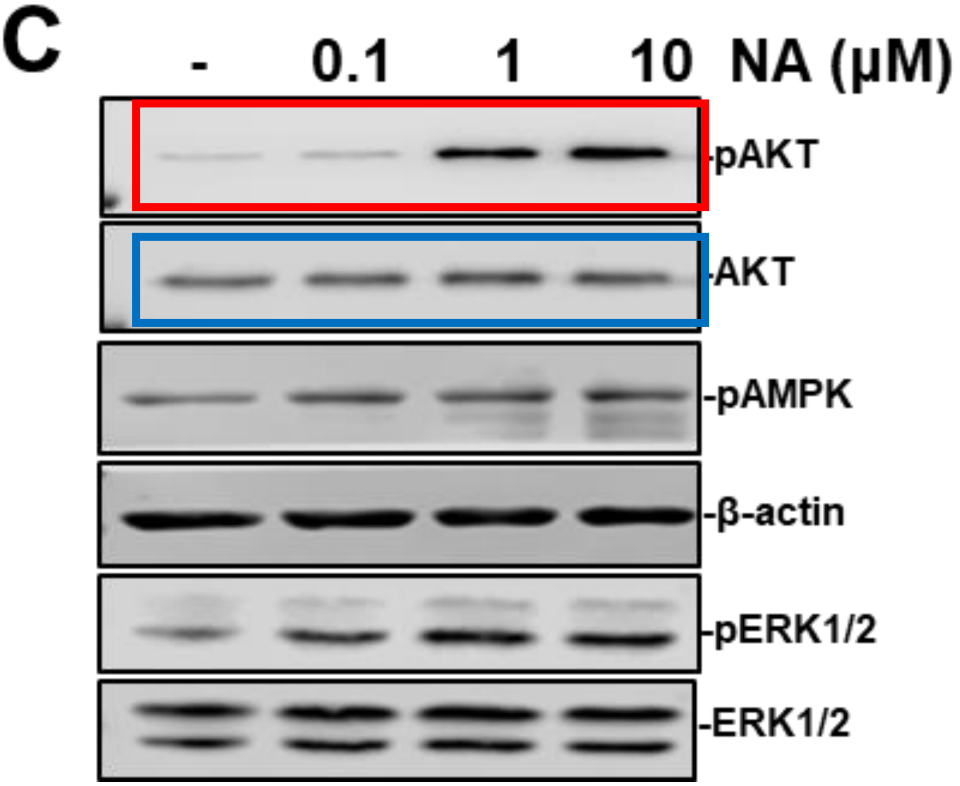

Supplementary Gel Images

FULL BLOT

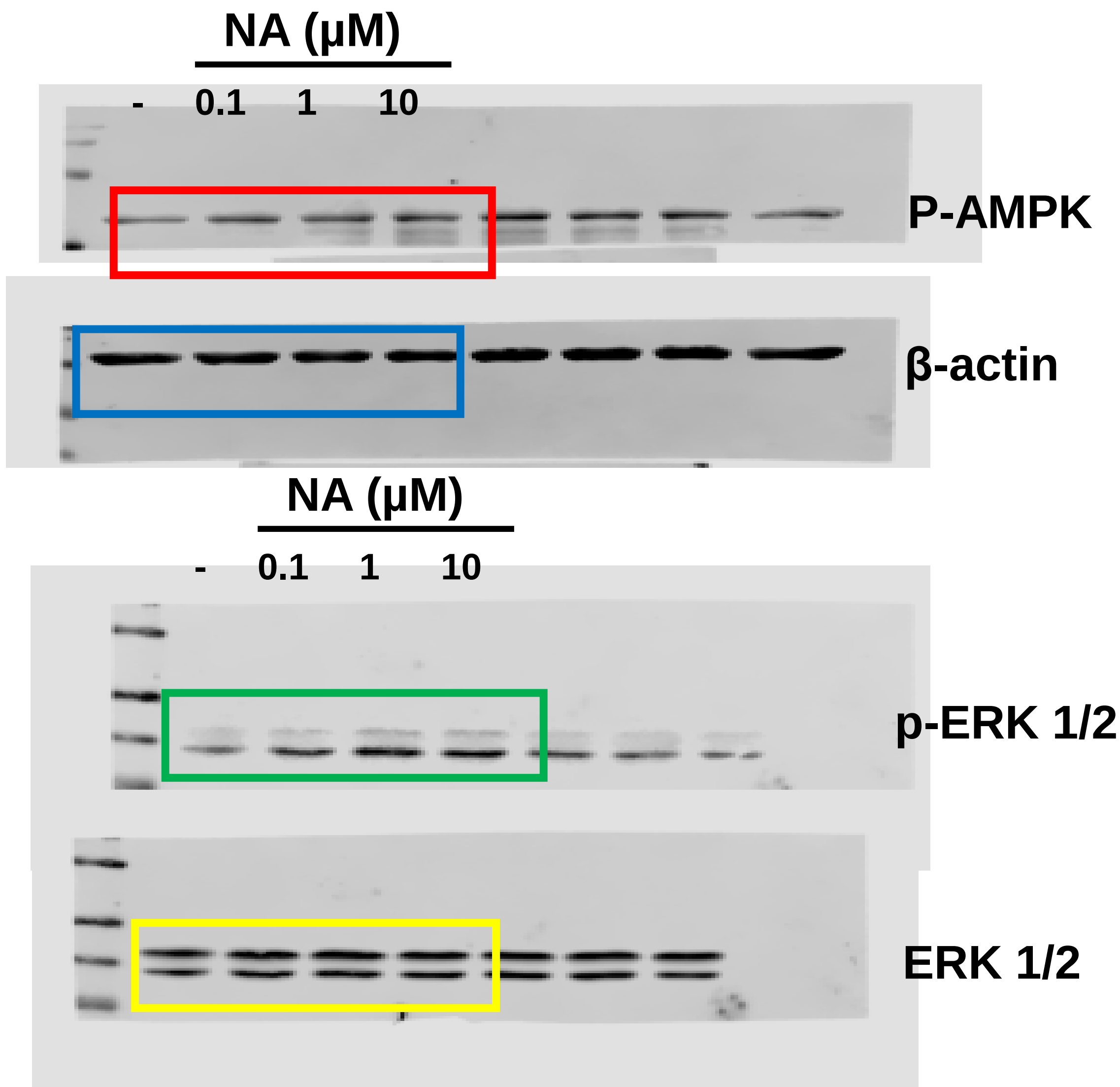

FIGURE 5 IN PAPER

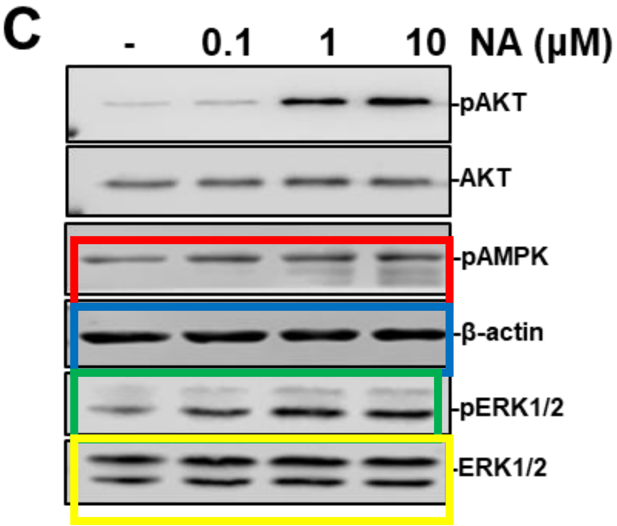

Supplementary Gel Images

FULL BLOT

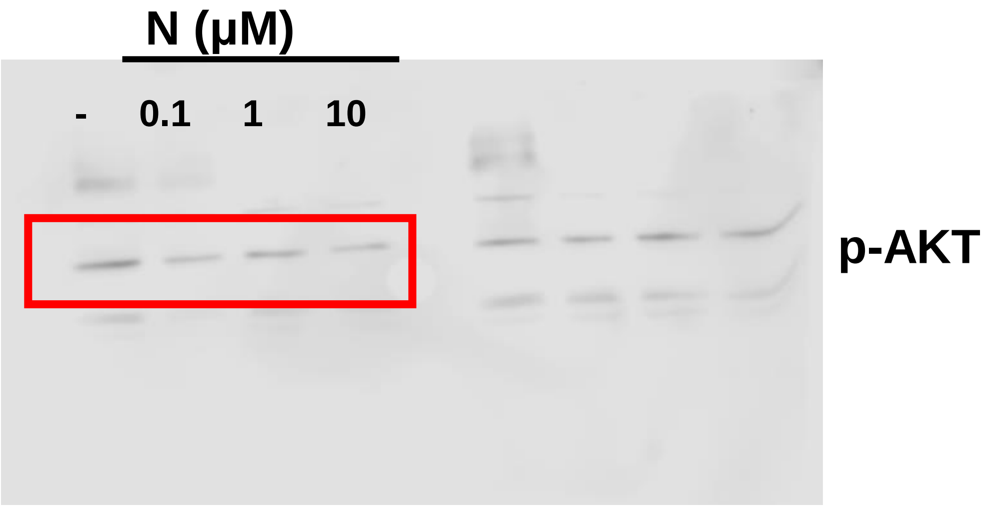

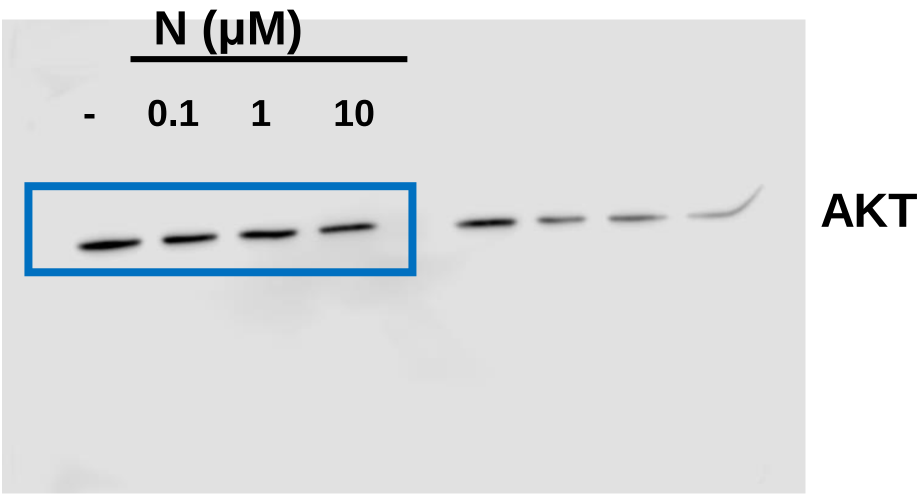

FIGURE 5 IN PAPER

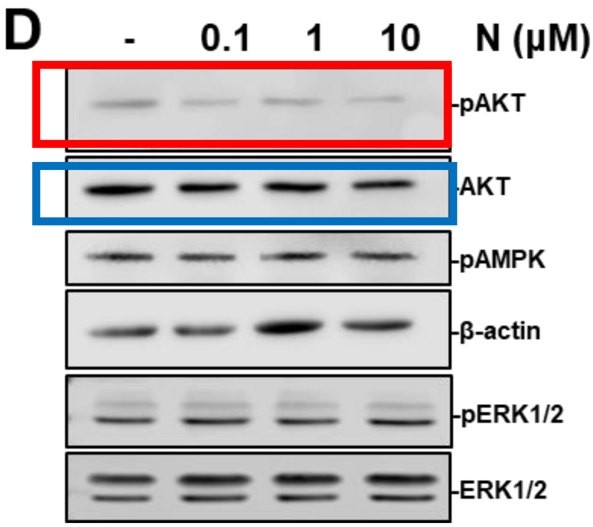

Supplementary Gel Images

FULL BLOT

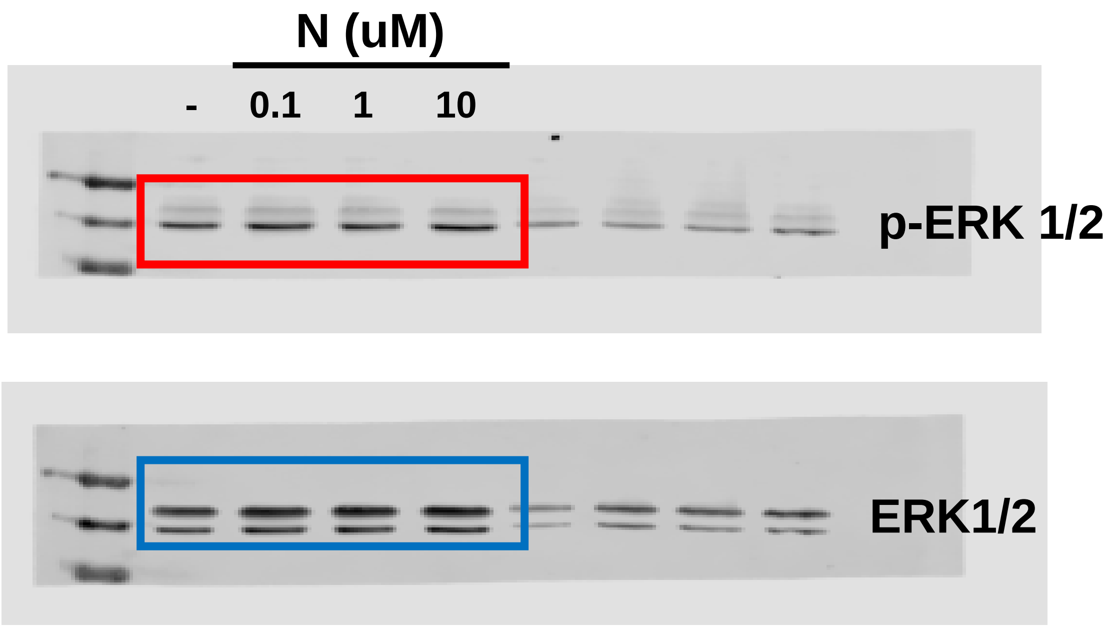

FIGURE 5 IN PAPER

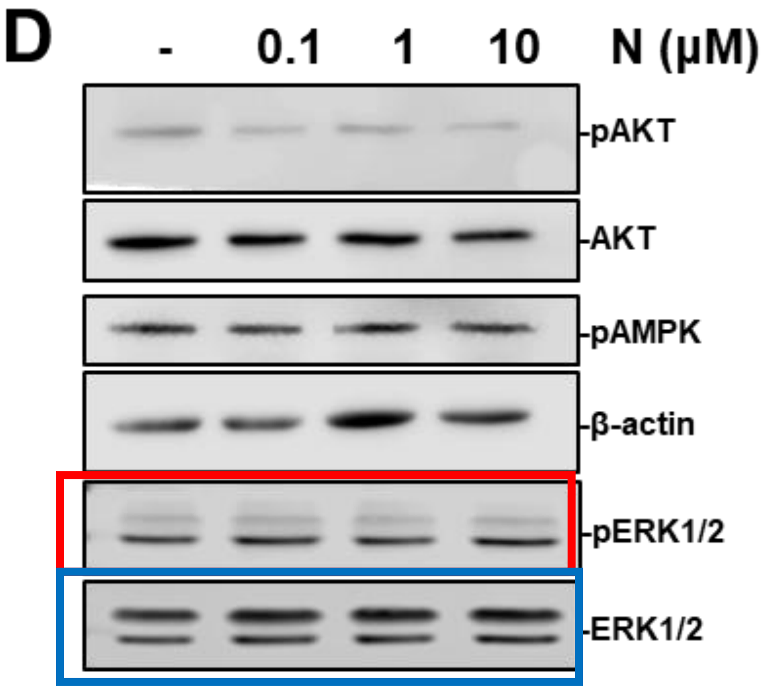

Supplement: Supplementary file 1 — Supplementary information. [file 41598_2020_69500_MOESM1_ESM.pdf]
